# Genetic Variation in the *TAS2R38* Bitter Taste Receptor and Smoking Behaviors

**DOI:** 10.1371/journal.pone.0164157

**Published:** 2016-10-06

**Authors:** Davide S. Risso, Julia Kozlitina, Eduardo Sainz, Joanne Gutierrez, Stephen Wooding, Betelihem Getachew, Donata Luiselli, Carla J. Berg, Dennis Drayna

**Affiliations:** 1 Laboratory of Communication Disorders, National Institute on Deafness and Other Communication Disorders, National Institutes of Health, Bethesda, Maryland, United States of America; 2 Laboratory of Molecular Anthropology and Centre for Genome Biology, Department of Biological, Geological, and Environmental Sciences, University of Bologna, Bologna, Italy; 3 McDermott Center for Human Growth and Development, University of Texas Southwestern Medical Center, Dallas, Texas, United States of America; 4 Health Sciences Research Institute, University of California Merced, Merced, United States of America; 5 Department of Behavioral Sciences and Health Education, Emory University Woodruff Health Sciences Center, Atlanta, GA, United States of America; Universita degli Studi di Pisa, ITALY

## Abstract

Common *TAS2R38* taste receptor gene variants specify the ability to taste phenylthiocarbamide (PTC), 6-n-propylthiouracil (PROP) and structurally related compounds. Tobacco smoke contains a complex mixture of chemical substances of varying structure and functionality, some of which activate different taste receptors. Accordingly, it has been suggested that non-taster individuals may be more likely to smoke because of their inability to taste bitter compounds present in tobacco smoke, but results to date have been conflicting. We studied three cohorts: 237 European-Americans from the state of Georgia, 1,353 European-Americans and 2,363 African-Americans from the Dallas Heart Study (DHS), and 4,973 African-Americans from the Dallas Biobank. Tobacco use data was collected and *TAS2R38* polymorphisms were genotyped for all participants, and PTC taste sensitivity was assessed in the Georgia population. In the Georgia group, PTC tasters were less common among those who smoke: 71.5% of smokers were PTC tasters while 82.5% of non-smokers were PTC tasters (P = 0.03). The frequency of the *TAS2R38* PAV taster haplotype showed a trend toward being lower in smokers (38.4%) than in non-smokers (43.1%), although this was not statistically significant (P = 0.31). In the DHS European-Americans, the taster haplotype was less common in smokers (37.0% vs. 44.0% in non-smokers, P = 0.003), and conversely the frequency of the non-taster haplotype was more common in smokers (58.7% vs. 51.5% in non-smokers, P = 0.002). No difference in the frequency of these haplotypes was observed in African Americans in either the Dallas Heart Study or the Dallas Biobank. We conclude that *TAS2R38* haplotypes are associated with smoking status in European-Americans but not in African-American populations. PTC taster status may play a role in protecting individuals from cigarette smoking in specific populations.

## Introduction

Tobacco smoking is a major worldwide health problem and is a leading cause of preventable disease [1–2].

Cigarettes and other tobacco products contain bitter compounds including nicotine, which contribute to the chemosensory properties of tobacco [3] and stimulate multiple sensory systems, including taste transduction pathways [4]. Since bitter taste has evolved to identify potentially toxic compounds [5], and thus protect against harmful foods, aversion to this taste may prevent smoking and nicotine dependence [6].

Receptors for human bitter taste are encoded by the *TAS2R* gene family which comprises 25 functional genes [7] and 11 pseudogenes [8] that have been subject to evolutionary forces [9–10–11–12]. The most studied gene in this family is *TAS2R38*, which encodes a receptor that mediates the ability to taste the bitter compounds phenylthiocarbamide (PTC) and 6-n-propylthiouracil (PROP) [13–14]. Two common forms of this gene exist worldwide, defined by amino acids at positions 49, 262 and 296 that constitute the PAV (Proline, Alanine, Valine, “PTC taster”) and AVI (Alanine, Valine, Isoleucine, “PTC non-taster”) haplotypes.

*TAS2R38* haplotypes have been hypothesized to influence smoking habits and nicotine dependence, since it has been shown that this gene has a lower expression in smokers, when compared to non-smoker individuals [15]. However, the results of previous studies have been conflicting. For example, a study examining both African-American (AA) and European-American (EA) individuals found a significant association between *TAS2R38* haplotypes and smoking, with the non-taster AVI haplotype being positively associated with smoking quantity and nicotine dependence. This was seen only in AA [16]. Another study analyzed German participants and found that individuals carrying the PAV taster haplotype smoked significantly fewer cigarettes per day [17]. In contrast, another study of individuals of European descent found no association between the PAV or AVI haplotypes and smoking. Moreover, this study found that the rare AAV haplotype was associated with a lower incidence of smoking [18]. In addition, a recent study investigated the relationship between *TAS2R38* haplotypes and menthol cigarette smoking and found that the PAV haplotype was associated with menthol cigarette use in pregnant female Caucasian smokers [19].

These mixed findings motivated the current study, which examined the association between *TAS2R38* PAV, AVI and rarer haplotypes and cigarette smoking in a larger number of individuals from three independent cohorts of both EA and AA individuals.

## Materials and Methods

### Research Participants

#### Georgia population

A total of 237 EA were chosen based on their tobacco product usage from a longitudinal study involving young adults attending seven Georgia colleges or universities [20]. Variables including sex, age and current smoking status were obtained from all participants during the web-based baseline survey in the fall of 2014; smoking status was also obtained again in Spring 2015. Individuals were defined as current smokers if they reported to have smoked in the past 30 days, as previously described [20–21]. In the spring of 2015, participants were sent an Oragene kit and a commercial taste-strip containing PTC (Thermo Fisher Scientific Inc., Catalog Number: S85287A) via mail with instructions regarding how to complete saliva provision and the taste-strip test; the responses for the latter were recorded by participants and sent back with the Oragene kit. Participants were defined as tasters if they categorized the taste of the PTC papers-strip to be “mild or strong” and as non-tasters if they reported “no taste”.

#### Dallas Heart Study

The Dallas Heart Study is a multiethnic population-based probability sample of Dallas County, Texas residents. The study design and recruitment procedures have been previously described in detail [22]. The original cohort was enrolled between 2000 and 2002, and all participants, as well as their spouses or significant others, were invited for a repeat evaluation in 2007–2009 (DHS-2). During each visit, participants completed a detailed survey including questions regarding demographics, socioeconomic status, medical history, and lifestyle factors (including tobacco use), and underwent a health examination. Ethnicity was self-assigned. A total of 2,363 AA and 1,353 EA DHS participants with available genotype and smoking phenotype data were included in the present study. Current smokers were defined as individuals who smoked at least 100 cigarettes in their lifetime and smoked on at least some days in the previous 30 days. Smoking quantity was defined as a categorical variable in all cohorts, sub-dividing smokers in three groups (less than 6 cigarettes per day, 6–19 cigarettes per day and 20 or more cigarettes per day).

#### Dallas Biobank

The Dallas Biobank is a repository of DNA and plasma samples from individuals ascertained at various locations in north-central Texas. The present study includes a total of 4,973 AA Biobank participants for whom the genotype and smoking phenotype data were available. Current smokers were defined as people who identified themselves as smokers and said they were currently using tobacco products.

### Ethical Statement

All participants were over 18 years of age and were enrolled with written informed consent. For the Georgia population, the study was approved by the Institutional Review Boards of Emory University, ICF Macro International, Albany State University, Berry College, University of North Georgia, and Valdosta State University. For the DHS and Biobank populations, the study was approved by the Institutional Review Board of the University of Texas Southwestern Medical Center.

### DNA Collection, Purification, and Sequencing

DNA from the Georgia population was collected using Oragene saliva collection kits and extracted according to the manufacturer’s protocol (Genotek Inc., Kanata, Ontario, Canada). The single coding exon of the *TAS2R38* gene was completely sequenced using dideoxy Sanger sequencing [23]. A dedicated set of primers modified from Kim et al., 2003, was adopted (Table A in S1 File) as previously published [24]. DNA chromatograms were analyzed and checked individually in order to evaluate the presence of calling errors with the Lasergene suite (DNASTAR, Madison, Wisconsin) [25]. In the DHS and the Dallas Biobank, genomic DNA was extracted from circulating leukocytes. A total of 4,597 DHS and 4,973 Biobank participants were previously genotyped using the Illumina Human-Exome BeadChip, which assayed the *TAS2R38 rs713598*, *rs1726866*, and *rs10246939* variants, residing in the codons for amino acid positions 49, 262, and 296 within the *TAS2R38* coding sequence. Genetic ancestry was estimated using EIGENSTRAT software [26].

### Statistical Analyses

Statistical analysis was performed using the R statistical analysis software [27]. Baseline characteristics of the study participants were compared using t-tests for continuous variables and chi-square tests for categorical variables. We used PLINK [28] to perform an initial quality control of genotypes and excluded variants with a call rate <90% or a deviation from Hardy-Weinberg equilibrium (HWE) (P<0.001). PHASE [29] was used to statistically infer *TAS2R38* haplotypes, using individuals from the 1000 Genomes Phase 1 [30] as a reference. Only haplotypes with posterior probability of 0.9 or above were considered for further analyses. Differences in *TAS2R38* haplotype distributions between smokers and non-smokers were explored using logistic regression in PLINK, with adjustments for demographic variables such as age, sex, the leading principal components of ancestry and the study indicator. An additive model was assumed for the effect of haplotypes. The significance levels of the association tests were adjusted using the Bonferroni correction in the Georgia cohort (adjusted P = P value X number of individual tests) and P<0.05 was considered statistically significant. For replication of the results in the DHS and Biobank populations, we reported the nominal p-values.

## Results

### Subject Cohort Demographics and Smoking Behaviors

Baseline and demographic characteristics of our study populations, stratified by cohort, are shown in Table 1. The average age of the individuals of the Georgia cohort was 20.91 +/- 1.95. Of the 237 participants, 123 (51.9%) were current smokers and the remaining 114 (48.1%) were non-smokers. No differences were found in the mean age of smokers (20.6) and non-smokers (21.2; P = 0.85). A higher, but not significant, percentage of smokers than non-smokers were female (54.4% of smokers versus 47.4% of non-smokers, P = 0.72). PTC sensitivity showed the classical bimodal distribution among participants, with 182 (76.8%) individuals classified as tasters and 55 (23.2%) as non-tasters. No significant age (P = 0.61) or gender (P = 0.34) differences were observed between PTC-taster and non-tasters.

**Table 1:** Characteristics of the study participants in the three different cohorts. DHS, Dallas Heart Study; AA, African-Americans; EA, European-Americans.

Characteristic	Georgia—EA	DHS—AA	DHS—EA	Biobank—AA
Number of participants	237	2363	1353	4973
Age, mean (SD)	20.9 (1.9)	48.2 (11.3)	50.1 (11.2)	44.8 (14.6)
Female, N (%)	121 (51.0%)	1410 (59.7%)	724 (53.5%)	3238 (65.1%)
Smokers, N (%)	123 (51.9%)	723 (30.6%)	316 (23.4%)	1526 (30.7%)
Smoking quantity*, N (%)				
≤5 cigs/day	91 (74.0)	211 (29.5)	47 (15)	715 (53.7)
6–19 cigs/day	28 (22.7)	318 (44.5)	116 (37.1)	554 (41.6)
≥20 cigs/day	4 (3.3)	186 (26)	150 (47.9)	63 (4.7)
n/r	/	8	3	194

*Smoking quantity was not available for some participants.

n/r–non-response.

The Dallas Heart Study population was significantly older than the Georgia cohort (mean age 48.2 and 50.1 years in AA and EA participants respectively, P<0.05). The proportion of female participants was slightly higher among DHS AA than EA participants (59.7% and 53.5%, respectively, P<0.05). DHS EA participants had a lower prevalence of smoking (23.4%) than those of either the Georgia cohort (51.9%, p <0.05) or the DHS AA participants (30.6%, P<0.05). Among DHS EA participants, smokers were on average 5 years younger than non-smokers (mean age 46.4 vs. 51.2 years, respectively, P<0.001, Table A in S1 File). There was no difference in age between AA smokers and non-smokers. In contrast to the Georgia cohort, we found a higher proportion of women among non-smokers in both ethnicities in DHS (64.2% vs. 49.4% in AA, P<0.001; 54.6% vs. 50.0% in EA, P>0.05).

The Dallas Biobank population was older than the Georgia population (mean age 44.8, P<0.001) but younger than both the DHS AA and EA participants (P<0.05). In addition, the Biobank included a higher proportion of females than either the Georgia or DHS AA/EA participants (65.1%, P<0.05). The fraction of individuals who were smokers was lower in this population compared to the Georgia cohort (30.7% vs. 51.9%, P<0.001) but similar to the DHS AA participants (P>0.05). In the Dallas Biobank population, smokers were slightly younger than non-smokers (mean age 43.8 vs. 45.3, P = 0.001). In addition, smokers had a lower percentage of females than non-smokers (51.7% vs. 71.0%, P<0.001, Table B in S1 File).

### Associations between *TAS2R38* haplotypes, PTC Tasting-Status and Smoking Behaviors

Haplotype phasing of the genomic DNA sequence data revealed five major haplotypes (AVI, PAV, AAV, AAI, and PVI), with frequencies of 53.4%, 41.6%, 4.4%, 0.4% and 0.2%, respectively, in the Georgia sample. In the DHS EA population, the frequencies of the three major haplotypes (PAV, AVI, and AAV) were similar to those in Georgia (53.2%, 42.5%, and 4.1%, respectively). The DHS AA carried a greater number of haplotypes: PAV (47.0%), AVI (32.6%), AAI (19.1%), AAV (1.1%) and PVI (0.3%). Similarly, the Dallas Biobank population showed higher frequencies of rare haplotypes: PAV (47.6%), AVI (32.3%), AAI (19.0%), AAV (1.0%), and PVI (0.1%). The distribution of *TAS2R38* haplotypes did not show any statistical differences between Biobank and DHS AA individuals and between DHS EA and Georgia EA individuals (Table B in S1 File).

As expected, the frequency of *TAS2R38* haplotypes and diplotypes differed between PTC-tasters and non-tasters in the Georgia cohort, where PAV was the predominant haplotype in PTC-tasters (95.3%) and rarely present in PTC non-tasters (4.7%) (P<0.001). Most of the PAV/PAV homozygotes in this cohort were PTC-tasters (98.1%) as opposed to non-tasters (1.9%) (P<0.001). In this EA cohort, PTC tasting abilities differed between smokers and non-smokers: 71.5% of smokers were PTC tasters, while 82.5% of non-smokers were PTC tasters (P = 0.03). The frequency of the *TAS2R38* PAV haplotype showed a trend toward a difference between smokers (38.4%) and non-smokers (43.1%), although this was not significant (P = 0.31) in this small group. We also noticed a possible trend toward a difference in the distribution of *TAS2R38* AVI haplotype between smokers and non-smokers (55.3% and 49.9% respectively, P = 0.29), but again this result was not significant in this small sample.

In the DHS EA cohort, the frequency of the taster PAV haplotype was lower in smokers (37.0%) than in non-smokers (44.0%) (P = 0.003). Conversely the frequency of the non-taster AVI haplotype was higher in smokers (58.7%) compared to non-smokers (51.5%) (P = 0.002). We did not find a difference in the frequency of the AAV haplotype between smokers and non-smokers (Table 2). In order to replicate this association in a sub-sample of individuals more comparable to the Georgia cohort in demographic characteristics, we repeated this analysis in DHS EA individuals <40 years of age (N = 272). The observed differences in *TAS2R38* haplotypes frequencies between smokers and non-smokers in this subgroup were very similar to those in the entire population (PAV haplotype frequency 35% in smokers vs 44% in non-smokers, P = 0.05; AVI haplotype frequency 60% in smokers vs. 51% in non-smokers, P = 0.06). None of the *TAS2R38* haplotypes differed in frequency between smokers and non-smokers in African Americans in either the DHS or Biobank populations (P’s>0.05).

**Table 2:** Distribution of *TAS2R38* haplotypes between smokers and non-smokers in the DHS population.

Haplotype	Frequency Smokers	Frequency Non-Smokers	P-value
**African Americans:**			
**PAV**	0.47	0.48	0.18
**AVI**	0.33	0.32	0.61
**AAI**	0.19	0.19	0.60
**AAV**	0.01	0.01	0.12
**European Americans:**			
**PAV**	0.37	0.44	0.003
**AVI**	0.59	0.51	0.002
**AAV**	0.04	0.04	0.620

Pooling the data for AA participants from the DHS and Biobank populations and EA participants from the DHS and Georgia populations confirmed our previous un-pooled analyses. In particular, combining the results by meta-analysis showed no association between *TAS2R38* haplotypes and current smoking in AA individuals (P>0.05). For the EA cohorts, we confirmed the associations found in the two independent cohorts with the PAV (P = 0.001) and the AVI (P = 0.001) haplotypes.

Lastly, a significant association was observed between the AVI haplotype and the prevalence of heavy smoking (>20 cigarettes per day) in DHS EA, in the same direction as with smoking status. This haplotype was in fact associated with higher prevalence of heavy smoking (P = 0.009). We also noted an opposite, although not significant, trend for the PAV haplotype (P = 0.08). No significant association was found in DHS AA or in the combined AA cohorts.

## Discussion

Although the hypothesis that variations in bitter taste receptor genes confer protection against cigarette smoking has long been of interest, previous findings have been conflicting [16–17–18]. We have therefore recruited a larger number of individuals from three independent cohorts to further explore this question.

Our results show a significant association between common *TAS2R38* haplotypes and smoking in EA: carriers of the taster PAV haplotype, and PTC tasters, were significantly less likely to be current smokers. Conversely, carriers of the non-taster AVI haplotype and PTC non-tasters were significantly more likely to be regular smokers. In contrast, in two large samples of AA including a total of more than 7,000 participants, we found no association between the major *TAS2R38* haplotypes and smoking status. These findings support the hypothesis that *TAS2R38* haplotypes play a role in modulating smoking behaviors, although the effects may be population-specific.

The reasons for the lack of consistency among the previous findings and across ethnic groups are not completely clear. One possibility is that taste plays a differential role as a motivation for smoking in individuals who are heavy tobacco users compared to those who smoke only occasionally. Indeed, the characteristics of participants included in previous reports were quite varied and different from individuals included in the current study. The earliest study exploring the correlation between *TAS2R38* haplotypes and smoking behaviors [18] examined 567 unrelated participants of European descent, comprising 384 smokers recruited from two smoking cessation trials. Although no significant associations were found between PAV/AVI haplotypes and the odds of smoking, the analysis of current smokers revealed a correlation between these haplotypes and the importance of the taste of cigarettes as a motive for smoking, as measured by the WISDM-68 taste/sensory processes scale [31].

A second study [16] enrolled both EA (N = 197) and AA (400) families of heavy smokers (defined as individuals who have smoked for at least the previous 5 years, and have consumed at least 20 cigarettes per day for the preceding 12 months). A significant correlation between the non-taster AVI haplotype and smoking quantity (cigarettes per day) was reported in AA, and the taster PAV haplotype was associated with lower smoking quantity. No significant associations, however, were observed in their EA participants. Lastly, a recent study [17] recruited 1,007 German individuals comprising 330 smokers with 10.9 mean cigarettes per day and showed that carriers of at least one PAV allele showed significantly lower cigarette smoking per day.

In contrast to these previous studies, which focused on relatively homogeneous populations of heavy smokers, the current study included participants from three demographically diverse cohorts (the Georgia cohort, the DHS cohort, and the Dallas Biobank cohort), in which the prevalence of smoking and nicotine dependence was much lower. In addition, only a small fraction of participants recruited in our cohorts reported smoking more than 20 cigarettes per day. Together, our cohorts contained a total of 1,590 EA and 7,336 AA participants. We fully replicated the results previously reported by Keller and colleagues [17] in our study of two different EA cohorts, the Georgia and DHS populations. In these cohorts, cigarette smokers had a lower percentage of PAV-carriers. In addition, this haplotype was also associated with smoking quantity (data not shown). Moreover, in the Georgia population, smokers showed a lower percentage of PTC-tasters (associated with the PAV haplotype) when compared to non-smokers. This also agrees with previous findings [32–33].

We failed to replicate the results of the family-based study reported by Mangold et al. 2008 In our two AA cohorts, neither PAV nor AVI haplotypes showed different frequencies between smokers and non-smokers. One possible explanation is that most of the individuals recruited in that study were heavy smokers, for whom nicotine dependence was a stronger motivator than taste. Finally, since the smoking data were based on self-report, it is possible that measurement error introduced a bias in our estimates. Nevertheless, this is the largest study to date to investigate the relationship between *TAS2R38* haplotypes and smoking in an ethnically diverse cohort.

Based on both previous and present data, we conclude that *TAS2R38* haplotypes appear to be factors contributing to smoking status in EA, with PAV haplotype carriers and PTC tasters less likely to be smokers. This finding has now been replicated in three independent cohorts, two cohorts in the present study and one in a previous report [17]. In addition, we noted a similar trend in a recent paper studying a large cohort (N = 1,319) of individuals of Caucasian origin [34]. In contrast, *TAS2R38* haplotypes are not good predictors of smoking behaviors in AA. The lack of *TAS2R38* haplotype association with smoking in AA may be due to potentially confounding factors, such as age, gender and ascertainment of smoking status. In the previous studies, in fact, the definition of individuals as “smokers” and/or “current” smokers was different, as was the average age of individuals and the percentage of females. Our study has several possible limitations, including different recruitment mechanisms and baseline characteristics of the cohorts and the fact that tobacco use was self-reported resulting in possible misclassification of smoking status. In addition, the number of cigarettes smoked is quite different in the examined cohorts, with the Georgia population being mainly composed of light smokers. This may have attenuated the association between TAS2R38 haplotypes and smoking in this cohort. However, our results support the hypothesis that *TAS2R38* haplotypes and the related ability to taste specific bitter compounds (such as PTC and PROP) influence smoking behaviors in EA. This does not appear to be true in AA populations. Future studies will need to address potentially confounding variables such as ascertainment of smoking status, population stratification, ethnicity, age and sex.

## Supporting Information

S1 File Table A. Characteristics of the Georgia, DHS and Biobank participants by smoking status. Table B. TAS2R38 Haplotype Frequencies in the Study Cohorts.
(DOCX)
