# Supplementary material for: Genetic Variation in the TAS2R38 Bitter Taste Receptor and Smoking Behaviors
Source: PLoS One. 2016 Oct 6;11(10):e0164157. doi: 10.1371/journal.pone.0164157 (PMC5053502; doi:10.1371/journal.pone.0164157)
Supplement: S1 File — Table A. Characteristics of the Georgia, DHS and Biobank participants by smoking status. Table B. TAS2R38 Haplotype Frequencies in the Study Cohorts. (DOCX) [file pone.0164157.s001.docx]

**Supporting Information File S1 for:**

**Genetic Variation in the *TAS2R38* Bitter Taste Receptor and Smoking Behaviors**

Davide S. Risso^1,2^, Julia Kozlitina^3^, Eduardo Sainz^1^, Joanne Gutierrez^1^, Stephen Wooding^4^, Betelihem Getachew^5^, Donata Luiselli^2^, Carla J. Berg^5^, Dennis Drayna^1*^

^1^Laboratory of Communication Disorders, National Institute on Deafness and Other Communication Disorders, National Institutes of Health, Bethesda, Maryland

^2^Laboratory of Molecular Anthropology and Centre for Genome Biology, Department of Biological, Geological, and Environmental Sciences, University of Bologna, Bologna, Italy

^3^McDermott Center for Human Growth and Development, University of Texas Southwestern Medical Center, Dallas, Texas

^4^Health Sciences Research Institute, University of California, Merced

^5^Department of Behavioral Sciences and Health Education, Emory University Woodruff Health Sciences Center, Atlanta, GA

* Corresponding author

E-mail: [drayna@nidcd.nih.gov](mailto:drayna@nidcd.nih.gov)

This S1 file includes:

Table A

Table B

**S1- Table A.** Characteristics of the Georgia, DHS and Biobank participants by smoking status.

| **Characteristic** | **Non-smokers** | **Smokers** | **P-value** |
| --- | --- | --- | --- |
| **Georgia cohort** |  |  |  |
| N | 114 | 123 |  |
| Age, years, mean (SD) | 21.2 (1.9) | 20.6 (1.8) | 0.85 |
| Female, N (%) | 54 (47.4%) | 67 (54.5%) | 0.72 |
|  |  |  |  |
| **DHS African American** |  |  |  |
| N | 1640 | 723 |  |
| Age, years, mean (SD) | 48.4 (11.6) | 47.6 (10.4) | 0.13 |
| Female, N (%) | 1053 (64.2%) | 357 (49.4%) | <0.001 |
|  |  |  |  |
| **DHS European American** |  |  |  |
| N | 1037 | 316 |  |
| Age, years, mean (SD) | 51.2 (11.3) | 46.4 (10.2) | <0.001 |
| Female, N (%) | 566 (54.6%) | 158 (50.0%) | 0.17 |
|  |  |  |  |
| **Biobank African American** |  |  |  |
| N | 3447 | 1526 |  |
| Age, years, mean (SD) | 45.3 (15.1) | 43.8 (13.4) | 0.001 |
| Female, N (%) | 2449 (71.0%) | 789 (51.7%) | <0.001 |

**S1- Table B.** *TAS2R38* Haplotype Frequencies in the Study Cohorts.

**
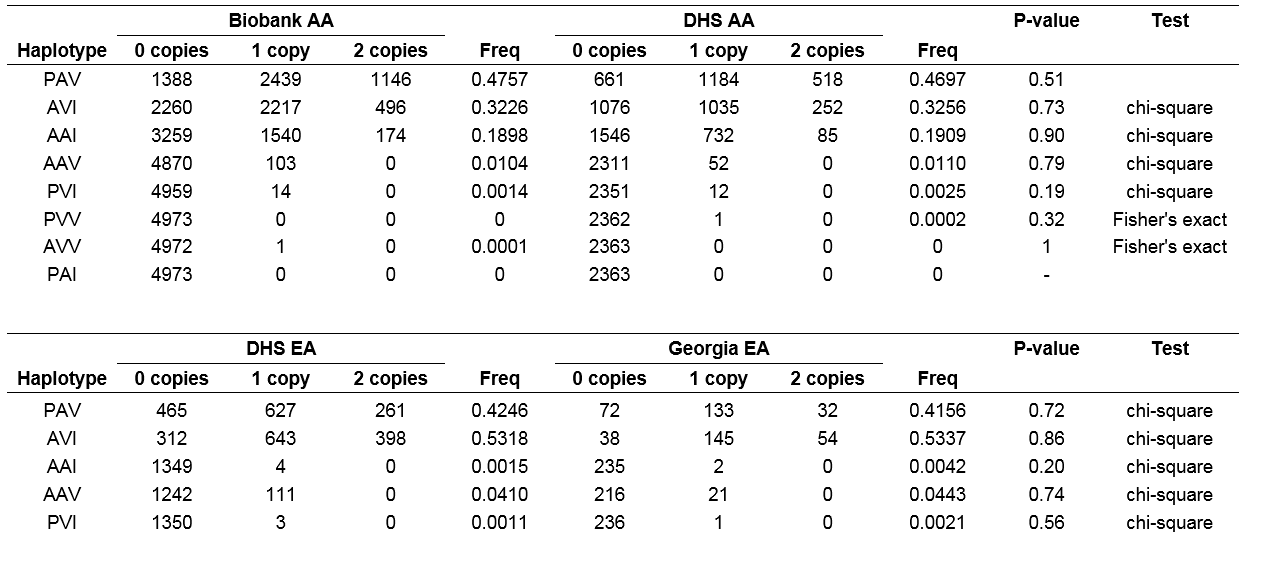
**
